# Research Progress on Sulphur Migration Characteristics in Coal Chemical Looping Combustion Processes

**DOI:** 10.3390/nano16120763

**Published:** 2026-06-17

**Authors:** Mei An, Pengfei Hao, Jianping Zhang, Shuli Bai, Ye Liu, Ziyan Dai, Guodong Zhang

**Affiliations:** 1 Jiangmen Key Laboratory of Synthetic Chemistry and Cleaner Production, School of Environmental and Chemical Engineering, Wuyi University, Jiangmen 529020, China; 2112523039@wyu.edu.cn (P.H.); 3223004811@wyu.edu.cn (J.Z.); wyuchembsl@126.com (S.B.); 3123002035@wyu.edu.cn (Y.L.); 3123005066@wyu.edu.cn (Z.D.); 2 Key Laboratory of Plant Nutrition and the Agri-Environment in Northwest China, Ministry of Agriculture, College of Natural Resources and Environment, Northwest A&F University, Yangling 712100, China

**Keywords:** coal chemical looping combustion, sulphur element, multifunctional oxygen carriers, sulphur tolerance

## Abstract

Coal chemical looping combustion (CLC) enables high-concentration CO_2_ capture with low NOx emissions. However, coal-derived sulphur species in the fuel reactor (FR) present severe challenges, including oxygen carrier (OC) poisoning and CO_2_ stream contamination. This study identifies coal-derived sulphur within the FR as the primary emission source and systematically characterises its release patterns during pyrolysis and gasification, comparing in-situ gasification CLC (IG-CLC) and chemical looping with oxygen uncoupling (CLOU). Coal sulphur distribution pathways and their governing factors are systematically investigated, followed by a comprehensive characterization of sulphur release behaviour during pyrolysis and gasification. We propose a novel perspective advocating a paradigm shift from passive sulphur tolerance to active in-situ sulphur capture through the rational design of Multifunctional Oxygen Carriers (MOCs). This review provides a comprehensive theoretical framework and practical guidelines for designing sulphur-resistant systems, thereby accelerating the industrial deployment of clean coal chemical looping technologies.

## 1. Introduction

### 1.1. Background and Objectives

Coal chemical looping combustion (CLC) achieves fuel conversion by transferring oxygen from an air reactor (AR) to a fuel reactor (FR) via a circulating oxygen carrier (OC) [1]. Because volatile species released during coal pyrolysis react rapidly with the OC, the solid–solid reaction between coal char and the carrier limits the overall system rate. To address this bottleneck, researchers have utilised coal char gasification and developed oxygen-releasing carriers [2].

In IG-CLC, gasifiers react with coal coke to enhance the conversion of C, H, and O elements in coal into intermediates such as CO and H_2_, thereby converting the slower solid–solid reaction into a faster gas–solid reaction. In contrast, chemical looping oxygen-uncoupling combustion (CLOU) technology employs gaseous oxygen released from OC within the FR to react directly with coal coke [3], thus resolving the issue of sluggish coal–coke reactions [4,5]. Slow coal–coke reaction rates have prompted extensive research, but sulphur release from coal poses a dual challenge: it degrades atmospheric environmental quality and CO_2_ purity [6], and it simultaneously impairs the activity of OC [7,8]. Therefore, in-depth investigation is required into the desorption, conversion, and redistribution of sulphur elements during coal CLC processes. Furthermore, studies indicate [9,10] that the key to achieving CO_2_ separation and in-situ desulphurization in coal CLC technology lies in increasing the proportion of sulphur entering solid-phase compounds while reducing the escape of gaseous sulphur [11,12]. Accurately understanding the release patterns of sulphur in coal, its gas–solid phase distribution, and the associated influencing factors is therefore essential for the design of sulphur-capturing OC, the implementation of in-situ desulphurization, and the control of sulphur pollution [13,14].

**Figure 1:**
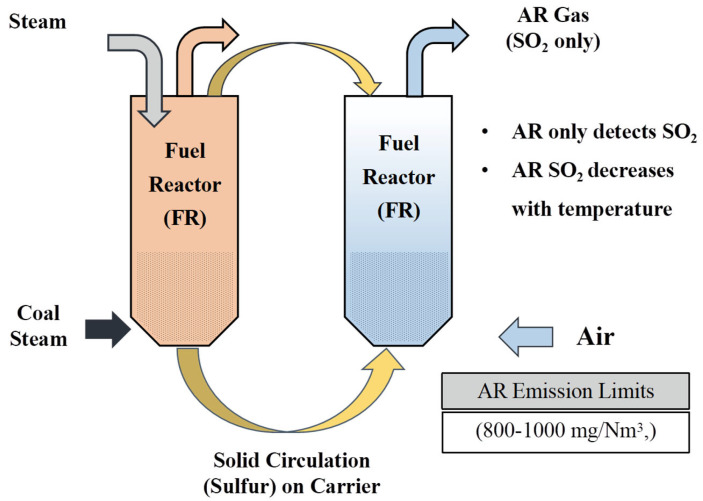
Schematic diagram of coal chemical looping combustion system (created by the authors).

This paper reviews the current research status of thermal desorption of sulphur and gasification desulphurization in coal. It systematically summarises the mechanisms governing sulphur release and distribution during coal CLC, outlines the pathways for sulphur distribution in coal, and identifies the key factors influencing the gas–solid distribution ratio of sulphur. The release characteristics of sulphur are further analysed in detail. Finally, prospects for new research directions related to the development of sulphur-capturing OC, the implementation of in-situ desulphurization, and the control of sulphur pollution are explored. Figure 1 shows a schematic diagram of the coal CLC system, clearly illustrating the core processes of the AR, FR and OC circulation.

### 1.2. Literature Search and Selection Methodology

To ensure the comprehensiveness, transparency, and reproducibility of this review, a systematic literature search and selection process was conducted. We searched three primary databases: Web of Science, Scopus, and Google Scholar, covering peer-reviewed journal articles published from 2000 to 2026. The search queries utilised combinations of keywords including: (“chemical looping combustion” or “CLC”) and (“sulphur” or “sulfur” or “H_2_S” or “SO_2_”) and (“migration” or “fate” or “transformation”) and (“coal” or “solid fuel”). The detailed inclusion and exclusion criteria, along with the database search results, are summarised in Table 1.

A total of 342 records were initially identified. After removing duplicates and screening titles and abstracts, 145 articles remained. Applying the strict inclusion and exclusion criteria (Table 1), 55 articles were finally selected for in-depth analysis. Articles focusing on unrelated topics, such as biodiesel, hydrate inhibition, and olive pomace oil, were strictly excluded to maintain a sharp focus on sulphur migration in CLC.

## 2. Patterns of Sulphur Precipitation

Coal reactions within the FR proceed through two distinct stages. Thermal decomposition initially triggers the release of volatile matter, followed by the combustion, gasification, and water–gas shift reactions of the residual coal coke. Sulphur release during the coal chemical looping process integrates the distinct emission patterns observed throughout both pyrolysis and coal coke gasification.

**Figure 2:**
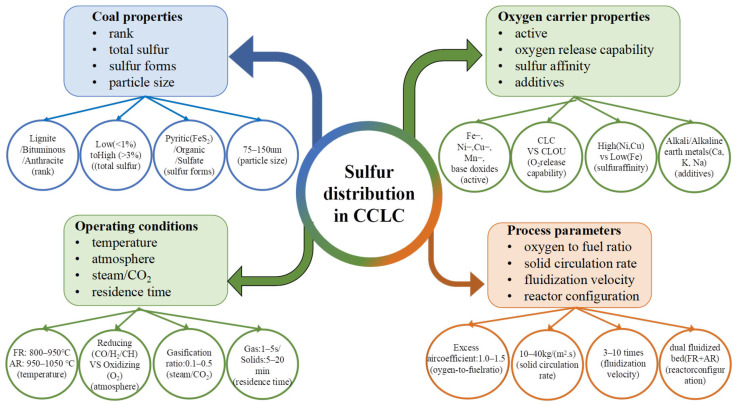
Schematic diagram of sulphur distribution and its influencing factors in coal chemical looping combustion. (created by the authors).

This study further outlines future research directions, specifically focusing on the development of sulphur-capturing OC, the implementation of in-situ desulphurization, and the mitigation of sulphur-related pollution. The sulphur distribution in CLC is determined by the synergistic effect of coal properties, OC characteristics, operating conditions, and process parameters. Figure 2 summarises the gas–solid distribution patterns of sulphur in coal CLC and the key influencing factors, providing an intuitive framework for the systematic analysis of sulphur migration behaviour. This figure summarises the core influencing factors and their effects on gaseous sulphur release and solid-phase sulphur fixation, laying a foundation for the discussion of sulphur migration and distribution in the CLC system.

### 2.1. Thermal Desorption Sulphur Patterns

Sulphur in coal is associated with organic matter within the coal and undergoes distribution and transformation during coal pyrolysis alongside the release of volatiles. Current research on thermally desorbed sulphur is extensive, encompassing the migration patterns of organic and inorganic sulphur in coal during pyrolysis, their mutual conversion processes, and the influence of pyrolysis atmospheres on the formation of sulphides in different forms.

#### 2.1.1. Sulphur Release and Transformation Mechanisms

Coal pyrolysis involves complex sulphur transformation and redistribution. Most sulphur remains within the semi-coke, whereas a smaller fraction volatilises alongside the gaseous products. Organic, pyritic, and sulphate sulphur species undergo complex chemical transformations, including mutual interconversion and the release of gaseous sulphur compounds [15,16]. Zhang et al. (2011) investigated sulphur transformation during brown coal pyrolysis using thermogravimetric analysis (TGA) [17], TGA-mass spectrometry (MS), and a fixed fluidised bed reactor, proposing a schematic mechanism for sulphur migration during coal pyrolysis. The decomposition of pyritic sulphur during coal pyrolysis generates reactive sulphur, which subsequently combines with reactive oxygen species in coal to form SO_2_. Organic sulphur decomposition initiates with the formation of sulphur-containing radicals. These intermediates subsequently undergo a series of complex reactions, ultimately yielding H_2_S, COS, CS_2_, and SO_2_. Certain sulphate species undergo decomposition to produce SO_2_. Sulphur species undergo simultaneous decomposition and release, while concurrently transforming into one another through complex interconversion pathways. For instance, reactive sulphur derived from pyrite decomposition combines with nascent semi-coke to form new organic sulphur compounds [18,19,20].

#### 2.1.2. Impact of Pyrolysis Atmosphere on Sulphur Speciation

Coal pyrolysis primarily generates H_2_S, COS, CS_2_, and SO_2_. Aliphatic and pyritic sulphur decomposition serves as the main source of H_2_S. Conversely, COS formation involves multiple pathways, including the decomposition of organic sulphur, the reaction between elemental sulphur (from pyrite pyrolysis) and carbon monoxide, and secondary interactions between hydrogen sulphide and carbon dioxide. Pyrolysis atmospheres significantly influence the generation of sulphur compounds [21]. Previous research demonstrates that atmospheres containing carbon monoxide and carbon dioxide promote carbonyl sulphide (COS) formation, particularly when compared to inert nitrogen environments [22]. Conversely, methane and hydrogen atmospheres, while enhancing H_2_S formation, inhibit the emission of other sulphur-containing gases. This phenomenon primarily arises because the reactive sulphur formed during pyrolysis readily captures hydrogen to form H_2_S. Current research on pyrolysis atmospheres mainly focuses on three categories: inert atmospheres, reducing atmospheres, and oxidising atmospheres [23].

Inert atmosphere pyrolysis primarily refers to pyrolysis conducted under non-reactive gas atmospheres, such as nitrogen, argon, or helium. Guo et al. (2014) employed a combined thermogravimetric-mass spectrometry (TG-MS) and pyrolysis-gas chromatography (Py-GC) approach to investigate the desorption and release behaviour of sulphur in model compounds under inert atmospheres [24]. They observed that the SO_2_ content in pyrolysis gases was significantly higher than that of H_2_S and COS. This was primarily attributed to the fact that the internal hydrogen content in an inert atmosphere was markedly lower than the internal oxygen content, leading most sulphur-containing radicals to readily combine with internal oxygen and escape in the form of SO_2_. Under inert conditions, unstable aliphatic sulphur compounds and cyclic sulphides decompose during pyrolysis to release H_2_S, whereas the more stable thiophenes exhibit thermodynamic resistance to decomposition.

Coal pyrolysis under reducing atmospheres encompasses both pure hydrogen and hydrogen-rich environments. Owing to the high reactivity of hydrogen, the hydrodesulphurisation of coal proceeds rapidly. Compared to inert atmospheres, the decomposition rate of pyrite during hydrogen pyrolysis is significantly enhanced. Research indicates that pyritic sulphur release rates exceeding 90% can be achieved during coal hydrogen pyrolysis. Li et al. (2021) proposed that the formation of sulphur-containing radicals from C–S bond cleavage in coal represents a key step in hydrodesulphurisation during hydrogen pyrolysis [25]. Through reaction field molecular dynamics simulations, Wang et al. (2015) proposed a reaction mechanism for the release of sulphur-containing gases from organic sulphur during coal hydrogenation pyrolysis [26]. They suggested that hydrogen weakens the C–S bonds in thiophenes, mercaptans, and sulphides, thereby reducing their bond-breaking energy and significantly enhancing the desulphurisation rate of coal sulphur during hydrothermal pyrolysis. Concurrently, hydrogen can generate hydrogen radicals, increasing the concentrations of gaseous products such as H_2_S, CH_4_, and H_2_O.

The pyrolysis process of coal in an oxidising atmosphere primarily refers to the thermal decomposition of coal in oxygen-containing gas environments. Sulphur in coal predominantly exists in the form of Fe–S and C–S bonds, forming chemical compounds with carbon; these bonds readily undergo thermal decomposition under pyrochemical conditions. Oxidising environments facilitate the cleavage of Fe–S bonds in coal-derived pyritic sulphur. This process triggers decomposition reactions that release gaseous species, including SO_2_, COS, and CS_2_ [27]. Oxygen-rich environments promote the oxidation of organic sulphur into gaseous SO_2_. Li et al. (2020, 2022) reported that the oxygen-induced cleavage of C–S bonds in coal generates a substantial quantity of sulphur-containing free radicals [28,29]. These radicals combine with hydrogen radicals to release H_2_S. They also react vigorously with oxygen-derived radicals, ultimately yielding SO_2_. This process effectively enhances the desulphurisation rate of organic sulphur in coal.

### 2.2. Sulphur Release Characteristics During Gasification

Regarding sulphur release during coal–coke gasification, researchers attribute this phenomenon to hydrogenation reactions between various sulphur species in coal and hydrogen produced during gasification [30]. Gasification atmospheres significantly dictate the sulphur reaction pathways during coal–coke gasification. Current research predominantly focuses on sulphur release under steam-based atmospheres [31], whilst studies examining sulphur release during gasification under carbon dioxide atmospheres remain scarce. Luo et al. (2014) investigated the formation patterns of gas-phase sulphides during steam–hydrogen gasification [32]. Direct evidence indicates that hydrogen sulphide (H_2_S) constitutes the primary product among gas-phase sulphides released during coal gasification, with COS and carbon disulphide (CS_2_) not detected. They propose that temperature and steam concentration are the direct factors influencing product distribution. Elevated temperatures facilitate the decomposition of sulphur-containing functional groups in coal, leading to a gradual increase in H_2_S content with rising temperatures. Concurrently, the reaction between steam and pyrite promotes hydrogen sulphide formation; thus, the concentration of H_2_S in the gas-phase sulphides correspondingly increases with rising H_2_O/C ratios. Zhao et al. (2010) investigated the effects of CO_2_ and steam atmospheres on sulphur release during coal coke gasification [33]. Analysis of gas-phase sulphide data via gas chromatography revealed that coal coke treated with CO_2_ as a gasifier released substantial COS into the gas phase with negligible H_2_S generation. Conversely, during steam gasification, sulphur from coal coke escaped into the gas phase primarily as H_2_S, with no detectable COS formation [34].

Sulphur release during coal gasification depends on the gasification atmosphere. Intrinsic factors, including coal rank and particle size, exert a significant influence on this process as well. Liu et al. (2017) investigated the effects of different sulphur forms and coal particle sizes on sulphur behaviour during gasification [35]. They found that inorganic sulphur and coal particle diameter affect gas mass transfer; a higher inorganic sulphur content or larger coal particle size reduces the release rate of gaseous products from inorganic sulphur decomposition. The synergistic release effect, driven by the mutual conversion between inorganic and organic sulphur, significantly influences sulphur emission profiles. Given the substantial variations in sulphur species distribution, coal samples containing both organic and inorganic sulphur exhibit considerably higher gaseous sulphide concentrations than those dominated by inorganic sulphur. However, these concentrations remain comparable to those observed in coals primarily composed of organic sulphur.

Both organic and inorganic sulphur in coal is released into the gas phase as the temperature increases and coal coke undergoes complete conversion. However, due to the differing thermal stabilities among various sulphur forms, their release temperature ranges also vary significantly. Notably, thermally stable organic sulphur compounds (e.g., thiophenes) and FeS decompose only at elevated temperatures. Some scholars have investigated the migration behaviour of pyrite during coal gasification processes [36]. They proposed that sulphur-bearing minerals in coal undergo a series of complex reactions, with a portion of sulphur released as hydrogen sulphide gas into the vapour phase. Another portion of the sulphur is absorbed and fixed by alkaline minerals within the coal, remaining in the coal ash. The more refractory pyritic sulphur and residual organic sulphur persist in the unburned coal coke, which ultimately exists as part of the coal ash.

### 2.3. Sulphur Release Mechanisms in IG-CLC and CLOU Processes

Sulphur release during coal CLC depends on multiple parameters, including coal rank, sulphur speciation, temperature, and reactor configuration. Furthermore, the gasifying agent, intermediate gasification products, and OC exert a critical influence on this process. This paper synthesises the mechanisms of sulphur release during CLC, categorised by thermal desorption and gasification-induced processes. In the IG-CLC process, the sulphur release pathway is uniquely modulated by the spatial-temporal evolution of the gas phase, which transitions from a highly reducing H_2_/CO rich zone near the coal feeding point to a CO_2_/H_2_O dominated zone near the OC bed. This dynamic atmosphere shift directly dictates the local selectivity of sulphur species, where the initial rapid hydrogenation yielding H_2_S is progressively superseded by the thermodynamic promotion of COS formation in carbon-rich zones. As the reaction advances, most sulphur in coal escapes in gaseous form, with residual undecomposed sulphur remaining in the coal ash. Compared to IG-CLC, the sulphur release mechanism differs slightly in the CLOU process, primarily due to variations in oxygen supply by the OC within the FR. Within the CLOU FR, the OC releases gaseous oxygen, transforming the atmosphere surrounding coal into an oxidising environment. The C–S bonds in coal are cleaved by oxygen, generating sulphur radicals. Upon reacting with hydrogen radicals and oxygen radicals, these sulphur radicals release substantial amounts of SO_2_ and H_2_S [37,38,39]. The oxidation of intermediate gases CO and H_2_ into CO_2_ and H_2_O alters the local chemical environment of sulphur within the coal matrix, which subsequently promotes the gradual formation of COS. Unreacted sulphur remains in the coal ash.

In general, both organic and inorganic sulphur in coal is released in gaseous form as temperatures increase, with the exception of certain refractory pyritic sulphur and relatively stable organic sulphur compounds, which persist in the coal ash. Gaseous sulphur compounds released during the IG-CLC process primarily consist of H_2_S and COS, with minor amounts of SO_2_. In contrast, the CLOU process predominantly yields SO_2_. The release of H_2_S is primarily governed by the extent of bonding between sulphur radicals and hydrogen atoms. Sulphur release patterns during coal CLC are inherently complex.

**Figure 3:**
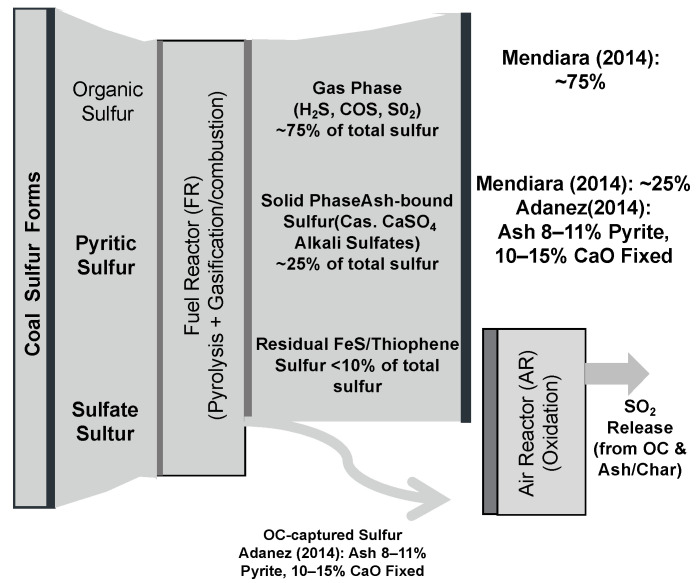
Quantitative Sankey diagram of sulphur distribution pathways during coal devolatilization and gasification in CLC (data normalised and adapted from typical bituminous coal experimental runs [40,41]) (created by the authors).

The reaction atmosphere undergoes continuous evolution, and the released sulphides interact dynamically with OC and coal ash. These factors present significant challenges for research, which explains the limited number of studies currently available in this field. Figure 3 shows a Sankey diagram illustrating the migration of sulphur during coal chemical loop combustion, providing a visual representation of the distribution of sulphur between the gas and solid phases and among different products.

Despite the extensive literature on coal sulphur evolution, a major scientific inconsistency lies in the decoupling of pyrite decomposition and organic sulphur release kinetics. Most existing models simplify coal pyrolysis as a homogeneous process, neglecting the heterogeneous mineral–organic matrix interactions. Thermodynamically, pyrite decomposes rapidly above 600 °C, but experimentally, the presence of organic matter often retards this process due to local mass transfer resistance and secondary capture by the carbonaceous matrix. This discrepancy highlights a critical research gap: the lack of in-situ, real-time diagnostic techniques capable of capturing the transient sulphur evolution at the micro-scale under high-heating-rate conditions typical of practical CLC reactors. Future research must focus on developing coupled quantum chemical and transport models to resolve these micro-scale inconsistencies.

## 3. Process Sulphur Distribution in IG-CLC and CLOU Processes

Coal sulphur undergoes significant transformation during CLC, resulting in the substantial release of gaseous sulphur species alongside the thermal conversion of the coal matrix [42,43]. These gaseous sulphides react with the OC and coal ash, with the majority escaping as gases from the reactor outlet. The remaining sulphur is retained in the OC and coal ash, which is then recycled into the AR and oxidised to release SO_2_.

**Figure 4:**
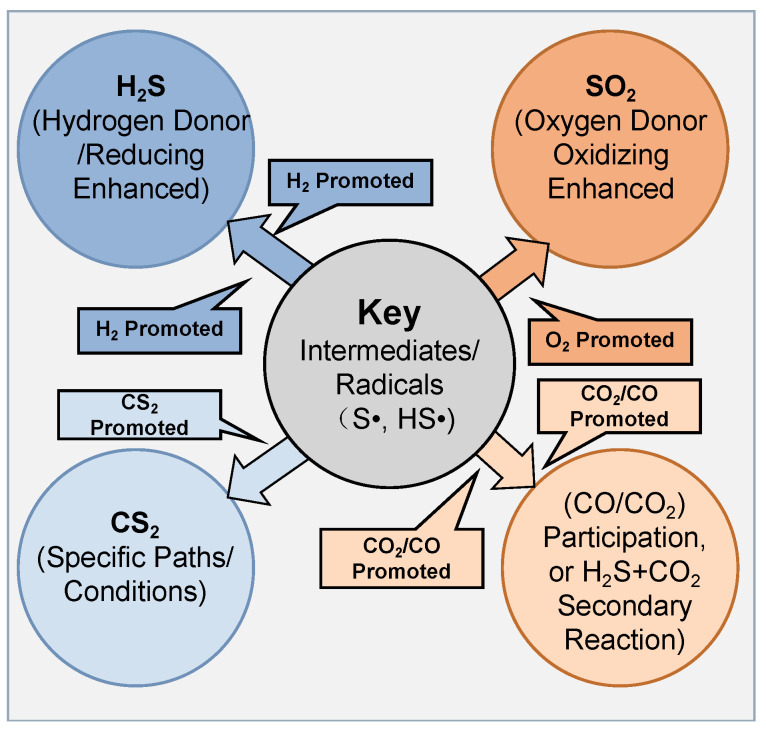
Reaction map of gas-phase sulphur species in CLC fuel reactor (created by the authors).

Drawing on the mechanisms of sulphur precipitation and redistribution observed in IG-CLC and CLOU processes. Two distribution processes occur throughout this stage: first, the distribution of sulphur between the gas phase and solid phase following coal pyrolysis and gasification; second, the redistribution of sulphur compounds in the gas phase. Specifically, within the FR, thermal desorption of sulphur occurs initially upon coal heating. Subsequently, under the action of gasifying agents, the sulphur remaining in the coal coke undergoes gasification and desorption, with a portion escaping as gaseous sulphur compounds. The remaining, less decomposable pyrite persists in the coal ash. Escaping gaseous sulphides interact with coal ash and OC, triggering a secondary distribution process. During this stage, OC facilitate the conversion of hydrogen sulphide into sulphur dioxide. Furthermore, reduced OC and alkaline minerals within the coal ash capture a portion of the gaseous sulphides, but the remaining unreacted species exit the reactor. To provide a holistic view of sulphur behaviour in coal CLC, the entire migration pathway—from solid coal to gas-phase species, and finally to solid-phase interactions with OC—is schematically consolidated in Figure 4.

## 4. Factors Influencing Sulphur Distribution in IG-CLC and CLOU Processes

Thermal conversion of coal sulphur within the FR produces four primary species: refractory sulphur, oxygen-carrier-absorbed sulphur, mineral-fixed sulphur, and gaseous sulphur emissions. The complex environment within the FR complicates the distribution of coal-derived sulphur. Furthermore, CLC technology remains at the laboratory research stage. Relevant studies on sulphur migration and redistribution have primarily focused on the influence of OC on sulphur distribution within coal, with limited research addressing coal rank, temperature, and OC/coal mass ratio.

### 4.1. Effect of Oxygen Carriers on Sulphur Distribution in IG-CLC and CLOU

**Figure 5:**
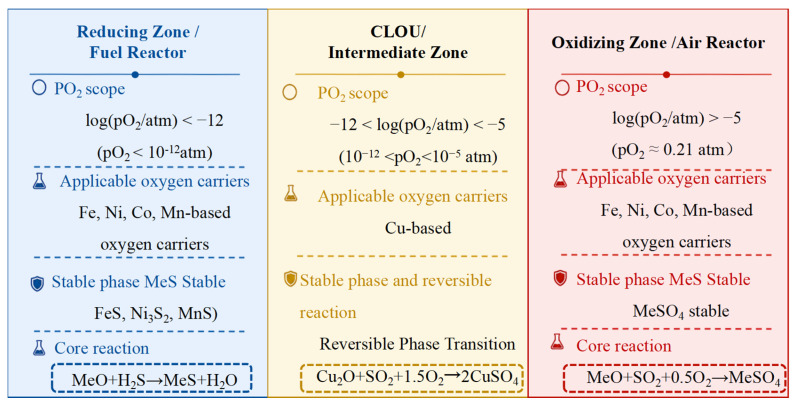
Interaction mechanism between oxygen carrier and sulphur compounds (created by the authors).

The OC acts not only as a thermal and oxygen vector but also as a reactive substrate that chemically alters sulphur distribution. On a macro scale, as demonstrated in pilot-scale operations, approximately 25% of coal sulphur is retained in the solid phase through interactions with OCs and ash minerals [44,45]. On a micro-mechanism level, this retention is governed by competitive gas–solid reactions. The gaseous sulphides H_2_S and COS released from coal are chemisorbed onto the active metal oxide sites Me_x_O_y_, where they are either oxidised to SO_2_ via lattice oxygen or fixed as metal sulphides Me_x_S_y_, such as Cu_2_S, Ni_3_S_2_, or Co_3_S_4_ on the reduced carrier surface. Unlike traditional coal combustion, the IG-CLC process utilises lattice oxygen from OC rather than gaseous oxygen. This substitution transforms the homogeneous reaction between gaseous sulphides and oxygen into a heterogeneous interaction between gaseous sulphides and the OC. Consequently, the OC plays a critical role in determining the distribution of gaseous sulphides. Once released within the FR, these sulphides adsorb onto the OC surface and undergo gas–solid reactions, which ultimately dictates the final sulphur distribution. Previous studies investigated gases such as H_2_S, SO_2_, COS, and CS_2_ generated from the reaction between the OC and coal using Fourier transform infrared spectroscopy (FTIR) under a nitrogen atmosphere in a thermogravimetric analyser (TGA) [46,47,48,49]. Through control experiments, they elaborated on the gas–solid reactions occurring between the OC and gaseous sulphides. They proposed that gaseous sulphides (H_2_S, COS, and CS_2_) released during coal pyrolysis and gasification are adsorbed onto the OC surface, where metal oxides facilitate their conversion into SO_2_ [50,51]. To further understand the effect of the OC on gaseous sulphur distribution, the residual solids after the reaction were characterised by X-ray diffraction (XRD) and X-ray fluorescence (XRF). Solid sulphides such as Co_3_S_4_, Ni_3_S_2_, MnS, and Cu_2_S were identified, and it was speculated that these solid sulphides are products of the reaction between H_2_S (one of the gaseous sulphides) and the reduced OC. To further elucidate the influence of OC on the distribution of gaseous sulphides, the authors employed HSC Chemistry software to perform thermochemical equilibrium calculations for the coal–OC reaction system. The results indicated that organic sulphur and inorganic sulphur in coal are converted into gaseous sulphides through a series of transformation reactions. Most of these gaseous sulphides are fixed by the OC and converted into solid sulphur, while only a small amount of sulphur is released in the form of gas, including SO_2_ converted by the OC. However, since thermochemical equilibrium calculations ignore the effects of temperature field and fluid field on gaseous sulphide distribution, the simulated theoretical data are inconsistent with the CLC process simulation data obtained by Mendiara et al. (2014) in a 100 Wth fluidised bed [40]. Figure 5 illustrates the mechanism of interfacial reactions between the OC and gaseous and solid-phase sulphur, and provides the key basis for understanding the sulphur fixation mechanism of the OC.

**Figure 6:**
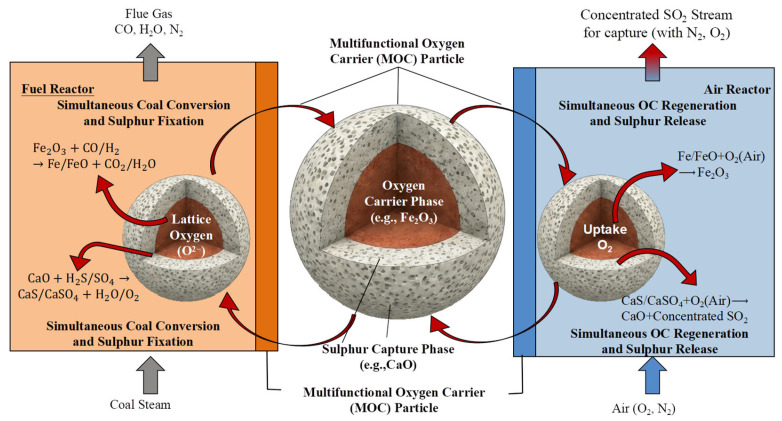
Schematic illustration of the simultaneous coal conversion, in-situ sulphur fixation, and OC regeneration mechanism using core-shell structured multifunctional OC (created by the authors).

To overcome the limitations of single-metal OC, the development of composite or multifunctional OC has attracted significant attention. A representative design is the core-shell structured OC, which combines the high oxygen-carrying capacity of iron oxides with the excellent sulphur-capturing performance of calcium-based sorbents. As schematically illustrated in Figure 6, during the reduction stage in the FR, the gaseous sulphur species released from coal gasification are immediately captured in-situ by the outer CaO shell to form CaS and CaSO_4_, while the inner Fe_2_O_3_ core provides lattice oxygen (O^2−^) for coal conversion. Subsequently, in the AR, the reduced iron core is re-oxidised to Fe_2_O_3_, and the sulphur-loaded shell releases concentrated sulphur species under oxidizing conditions, regenerating the active CaO phase. This synergistic mechanism effectively prevents sulphur contamination in the FR flue gas and facilitates downstream sulphur recovery.

Within the fuel reactor of CLOU, gaseous oxygen released by the OC distributes around sulphur in coal, placing the sulphur elements in an oxidizing atmosphere. Consequently, the OC not only influences the gas–solid phase distribution during the release of sulphur from coal but also affects the distribution of sulphur compounds in the gas phase. Adánez et al. (2014) observed that, in a circulating fluidised bed under a nitrogen atmosphere, oxygen released from the OC facilitates the oxidation of pyrite sulphur and the cleavage of C–S bonds in organic sulphur compounds [41], thereby promoting the substantial release of SO_2_. As the reaction progresses, the released gaseous oxygen further converts gases such as H_2_S and COS into SO_2_. Consequently, the gaseous product released at the FR outlet in the CLOU system is predominantly SO_2_.Furthermore, as the OC and gaseous sulphides coexist in the same reactor throughout the reaction, the OC may also exert an influence on the distribution of gaseous sulphides. Perez et al. (2016) investigated the reactivity of oxygen-releasing OC with SO_2_ using thermogravimetric analysis [44]. They observed that under a nitrogen atmosphere containing 8000 ppm SO_2_, the OC converted gaseous SO_2_ into solid copper sulphate. Conversely, in a fluidised bed reactor system under a nitrogen atmosphere containing 3000 ppm hydrogen sulphide, gaseous oxygen released from copper-based OC oxidised H_2_S to SO_2_. Subsequently, the oxygen-depleted copper-based OC adsorbed the hydrogen sulphide gas and converted it into solid Cu_2_S.

Typically, OC consist of an active component, a support material, and additives. In addition to the lattice oxygen and gaseous oxygen provided by the active component that influence sulphur distribution, the additives in the OC also exert an impact on sulphur allocation. Tian et al. (2017) observed in a small-scale fluidised bed that the addition of calcium oxide to copper-based OC significantly alters the distribution of gaseous sulphides during the CLOU process [52]. This process immobilised the majority of SO_2_ gas on the OC, thereby substantially reducing the release of gaseous SO_2_.

A critical analysis of the literature reveals a profound conflict between thermodynamic feasibility and kinetic viability regarding OC sulphur tolerance. For instance, while Fe-based OCs are thermodynamically predicted to form stable FeS in reducing atmospheres, experimental studies frequently report that the actual sulphur capacity is far below the theoretical limit due to surface pore blockage caused by rapid carbon deposition. Conversely, Ca-based OCs exhibit excellent thermodynamic affinity for sulphur capture, yet they suffer from severe sintering and mechanical degradation over multiple redox cycles, a challenge that remains unresolved in long-term continuous operations. The scientific gap here is the insufficient understanding of the dynamic evolution of the OC active surface under fluctuating redox states. Future efforts must transition from static thermodynamic screening to dynamic, multi-cycle kinetic characterization of OCs under realistic, sulphur-laden atmospheres.

### 4.2. Influence of Coal Rank and Total Sulphur Content on Sulphur Distribution

The contents of pyrite and thiophene in coal directly influence the sulphur remaining in coal ash during thermal conversion [53], thereby affecting the gas–solid distribution of sulphur. A higher total sulphur content in coal correlates with an increased pyrite content, while higher coal ranks are associated with greater amounts of stable thiophene-type organic sulphur. Wang et al. (2016) investigated the reaction between high-sulphur coal and OC under a nitrogen atmosphere using thermogravimetric analysis [49].

They found that refractory pyrite (FeS) and thiophene-type organic sulphur in high-sulphur coal remained in the coal ash, leading to an increase in the solid sulphur content. Tian et al. (2017) compared the sulphur distribution during the CLOU process between low-sulphur anthracite and high-sulphur lignite [52]. The data indicated that, compared to high-sulphur coal, low-sulphur coal released less sulphur in gaseous form, while a larger proportion of sulphur persisted in the coal ash as solid sulphides and sulphates.

### 4.3. Effect of Temperature on Sulphur Distribution

Temperature is the primary external factor influencing sulphur distribution in coal chemical looping processes. As temperature increases, the degree of coal reaction in the FR progressively intensifies. Elevated temperatures facilitate the release of volatile matter and the precipitation of sulphur from coal coke, thereby increasing the amount of gaseous sulphides released from coal and influencing the gas–solid distribution during coal sulphur precipitation [52]. Meanwhile, the physicochemical properties of OC are relatively sensitive to temperature, which means that temperature affects the reaction between OC and gaseous sulphides. Mendiara et al. (2014) investigated the CLC process using ilmenite-based OC in a fluidised bed [40].

**Figure 7:**
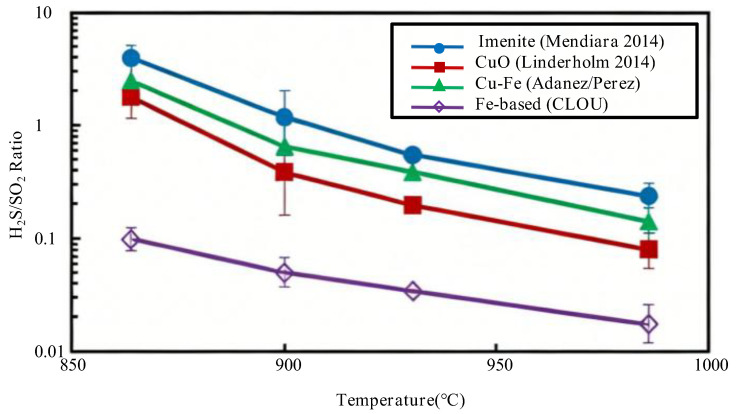
Effect of reaction temperature on H_2_S/SO_2_ ratio in CLC system (created by the authors) [40,41,44,54].

They observed a gradual decrease in the H_2_S/SO_2_ ratio with increasing temperatures. The authors attributed this phenomenon to the promotion of the reaction between H_2_S and the OC by high temperatures, thereby reducing the H_2_S content in the system while increasing the SO_2_ concentration. Figure 7 illustrates how temperature influences the H_2_S-to-SO_2_ ratio within the CLC system, providing a temperature-based basis for optimising the sulphur form.

### 4.4. Effect of Oxygen Carrier-to-Coal Ratio on Sulphur Distribution

**Figure 8:**
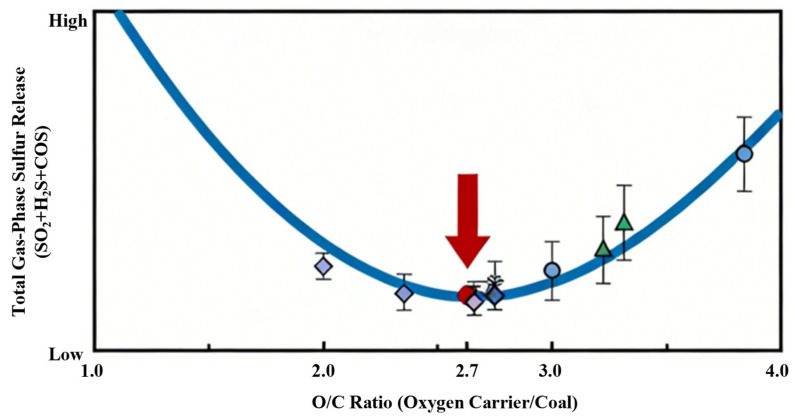
Effect of oxygen carrier/coal mass ratio (O/C ratio) on gas-phase sulphur release (created by the authors).

The OC/coal mass ratio directly influences the extent of coal conversion by the OC. Wang et al. (2016) thermodynamically modelled sulphur element conversion during the coal CLC process, concluding that the OC/coal mass ratio affects sulphur element distribution [49]. An increase in this ratio reduces the proportion of sulphur allocated to solid-state products. Tian et al. (2017) investigated the effect of the OC/coal mass ratio on sulphur distribution in a copper-based CLOU process [52]. They found that when the OC/coal mass ratio was 2.7, the minimum amount of gaseous sulphides was released per unit mass of coal, with the corresponding maximum amount of sulphides immobilised in the solid phase. Figure 8 shows that the mass ratio of OC to coal directly influences the amount of sulphur released into the gas phase, and that there is an optimal range for the solid–sulphur ratio. To provide a rigorous, quantitative evaluation of various OCs in coal CLC processes under sulphur-containing atmospheres, Table 2 compiles key performance metrics. This comparison systematically focuses on active phases, optimal operating temperatures, sulphur retention efficiencies in the solid phase, cyclic stability, and their corresponding thermodynamic behaviours.

## 5. Sulphur Release Characteristics in IG-CLC and CLOU Processes

The atmospheres surrounding coal within the FRs of IG-CLC and CLOU differ, resulting in distinct sulphur release characteristics. Within the IG-CLC FR, H_2_S and SO_2_ constitute the primary gaseous sulphide components. During the CLOU process, oxygen released from the OC oxidises sulphur-containing gases such as H_2_S to SO_2_. Table 3 summarises the fuel sulphur release characteristics for the IG-CLC and CLOU processes. Adánez et al. (2014) and Perez et al. (2016) observed in a 1.5 kWth apparatus that during the CLOU process using different copper-based OC and fuel coals [41,44], the majority of sulphur in the coal was released as SO_2_ from the FR outlet. In contrast, Mendiara et al. (2014) and Linderholm et al. (2014) using 100 kWth and 500 kWth apparatuses [40,54], observed that the outlet gas from the FR contained only SO_2_ and H_2_S during the in situ CLC process of fuel coal over ilmenite OC.

The primary sources of sulphur-containing gases in the AR are sulphides present in coal coke and sulphur compounds in coal ash. Experimental results indicate that only SO_2_ was detected in the outlet gas from the ARs of IG-CLC and CLOU systems. Furthermore, SO_2_ release from the ARs decreased with increasing temperature, primarily because elevated temperatures enhanced carbon conversion rates in the FR, thereby reducing the amount of coal coke and coal ash recycled into the AR. Some researchers observed that sulphur dioxide concentrations released from the AR exceeded emission standards [43,44], primarily due to pyrite concentrated in coal coke or coal ash. To investigate the influence of pyrite in coal on sulphur release characteristics in the AR during the CLOU process, Adánez et al. (2014) compared the S/C ratios in volatiles from coal pyrolysis and gases released during coke combustion [41]. They observed that carbon combustion and pyrite oxidation are competing reactions, with carbon combustion occurring first. This prevents most pyrite in coal from decomposing, leading to its enrichment in the coke. Meanwhile, sulphides immobilised in coal ash by CaO are recycled into the AR alongside coal ash and coke, undergoing decomposition reactions that release substantial amounts of SO_2_. Consequently, elevated concentrations of sulphur dioxide gas are present in the AR.

From a systems engineering perspective, managing sulphur migration in CLC involves a critical trade-off between sulphur retention in the FR and sulphur slip to the AR. Current literature often treats the FR and AR as isolated units, failing to address the loop-wide sulphur balance. If sulphur is successfully retained in the FR as solid sulphides, it is inevitably transported to the AR via the circulating solids, leading to SO_2_ emissions that compromise the carbon capture purity. If sulphur is allowed to gasify in the FR, it poisons the OCs and contaminates the syngas. This unresolved technological challenge requires a paradigm shift in reactor design. We propose that future research must investigate integrated multi-stage fluidised bed configurations with dedicated in-situ desulphurization zones, alongside the development of robust gas–solid separators to prevent the cross-contamination of sulphur species between the two reactors.

## 6. Conclusions

Since the inception of CLC for coal conversion, its inherent advantages—namely energy-efficient CO_2_ separation and high combustion efficiency—have gained global recognition. Extensive research has been dedicated to the impact of coal-derived sulphur on this technology, revealing that sulphur species compromise OC reactivity, degrade ambient air quality, and complicate CO_2_ storage and transportation. To address these challenges, technical strategies for in-situ CO_2_ separation and desulphurization have been proposed. By systematically analysing sulphur precipitation and distribution patterns, this study posits that the realization of in-situ desulphurization relies on the development of multifunctional OC (MOCs) capable of simultaneous sulphur capture and oxygen transport. Guided by the chemical looping principle, these MOCs facilitate coal conversion and sulphur fixation in the FR. Concurrently, they release captured sulphur and regenerate lattice oxygen within the AR.

This review has synthesised the critical pathways of sulphur migration and its interaction with OCs during coal CLC. To transition these laboratory-scale findings into viable industrial applications, several key innovations must be realised in the near future. The development of next-generation, sulphur-resistant composite OC is paramount. Future research should focus on the rational design of multi-metal oxides doped with alkali or alkaline earth metals, which can simultaneously promote fuel conversion and suppress sulphur-induced sintering or phase deactivation. The integration of advanced in-situ characterization techniques is highly needed. Utilizing synchrotron radiation, in-situ XRD, and environmental TEM during the CLC reaction will allow researchers to observe the real-time, nanoscale phase transitions of OCs under sulphur-rich atmospheres, providing direct kinetic data to refine predictive models. Finally, future industrial innovations must prioritise system-level integration. This includes coupling CLC reactors with downstream sulphur recovery units and optimizing the hydrodynamics of dual fluidised bed systems to minimise sulphur slip into the AR. Addressing these challenges through cross-disciplinary collaboration will be pivotal in driving the commercialization of clean, low-carbon coal CLC technology.

## Figures and Tables

**Table 1:** Literature search database, keywords, and selection criteria.

Parameter	Details
Databases	Web of Science, Scopus, Google Scholar
Time Span	2000–2026
Search Query	TS = ((“chemical looping” OR “CLC”) AND (“sulphur” OR “sulfur” OR “H_2_S” OR “SO_2_”) AND (“migration” OR “fate” OR “transformation” OR “interaction”) AND (“coal” OR “solid fuel” OR “oxygen carrier”))
Initial Records Identified	N = 342 (Web of Science: 156, Scopus: 124, Google Scholar: 62)
Inclusion Criteria	Peer-reviewed journal articles or high-quality conference proceedings written in English. Focus on sulphur transformation, migration pathways, or oxygen carrier sulphur poisoning in CLC systems. Studies involving coal, biomass, or solid fuel chemical looping processes.
Exclusion Criteria	Patents, book reviews, editorial materials, or duplicate publications. Studies focusing on conventional coal combustion or gasification without chemical looping. Studies on unrelated topics (e.g., biodiesel, hydrate inhibition, olive pomace oil, methane coupling).
Final Selected Articles	N = 55

**Table 2:** Comparison of sulphur migration characteristics, retention performance, and key mechanisms of typical oxygen carriers in coal chemical looping combustion.

Oxygen Carrier Type	Active Phase	Optimal Temp. Range (°C)	Sulphur Retention in Solid Phase (%)	Cyclic Stability (Cycles/Activity Retention)	Main Sulphur Product & Limitation	Ref.
Fe-based	Fe_2_O_3_/FeTiO_3_	960–1000	<1%	Excellent (700–800 h lifetime)	SO_2_ (75%) and H_2_S (25%); limited by lack of thermodynamic sulphur affinity.	[54]
Cu-based	CuO/SiO_2_	800	~31.4%	1 cycle	SO_2_, H_2_S limited by low melting point of Cu and Cu_2_S formation	[38]
Ca-based	CaSO_4_	900	41.8–64.6%	5 cycles	SO_2_, H_2_S particle sintering and sulphur depletion	[55]
Mn-based	MnFe_2_O_4_	900	~80%	Incomplete regeneration due to irreversible side reactions	Solid MnSMnS and MnSO_4_ limited by manganese silicate (Mn_2_SiO_4_) formation	[47]
Ni-based	NiO/Al_2_O_3_	950	~31.3%	1 cycle	SO_2_, H_2_S limited by sulphur poisoning (Ni_3_S_2_, NiS)	[38]

**Table 3:** Sulphur release characteristics of process fuels in IG-CLC and CLOU processes.

Coal Type	Total Sulphur (%)	Capacity (kW_t_)/Process/Gasifier/Oxygen Carrier	Air Reactor SO_2_ Concentration (mg/Nm^3^, Condition)	Fuel Reactor Sulphur Gas Species	Ref.
Lignite	5.2	0.5/CLC/H_2_O/Ilmenite	450 (930 °C, 9% O_2_)	H_2_S/SO_2_ = 0.33	[24]
Anthracite	0.8	100/CLC/H_2_O/Ilmenite	—	H_2_S/SO_2_ = 0.33	[32]
Lignite	5.2	1.5/CLOU/N_2_/Cu–Fe mixed oxides	800 (930 °C, 6% O_2_)	95% SO_2_	[25]
Lignite	5.2	1.5/CLOU/N_2_/CuO	1000 (935 °C, 6% O_2_)	95% SO_2_	[26]

## Data Availability

No new data were created or analysed in this study.
